# Circulating natural antibodies to inflammatory cytokines are potential biomarkers for atherosclerosis

**DOI:** 10.1186/s12950-018-0199-2

**Published:** 2018-11-16

**Authors:** Peng Wang, Huan Zhao, Zhenqi Wang, Xuan Zhang

**Affiliations:** 1 Jilin Provincial Key Laboratory on Molecular and Chemical Genetics, Second Hospital of Jilin University, 218 Ziqiang Street, Changchun, 130041 China; 2 School of Public Health, Jilin University, Changchun, 130021 China

**Keywords:** Atherosclerosis, Natural antibodies, Inflammatory cytokines, ELISA

## Abstract

**Background:**

Inflammatory cytokines contribute to the development of atherosclerosis. Natural antibodies in the circulation have protective effects on common diseases including atherosclerosis-related conditions.

**Purpose:**

The present study aimed to investigate the possible involvement of circulating IgG antibodies against inflammatory cytokines in atherosclerosis.

**Methods:**

A total of 220 patients with diagnosis of atherosclerosis and 200 healthy controls were recruited. Seven linear peptide antigens were used to develop an enzyme-linked immunosorbent assay in-house for detection of plasma IgG antibodies against interleukin 1β (fragments IL1β-1 and IL1β-2), IL6, IL8, tumor necrosis factor alpha (fragments TNFα-1 and TNFα-2) and IL1α.

**Results:**

Atherosclerotic patients had an increase in the levels of circulating IgG to TNFα-1(adjusted r^2^ = 0.038, *p* < 0.001) and IL1α (adjusted r^2^ = 0.025, *p* = 0.002) compared with control subjects. Female patients mainly contributed to increased anti-TNFα-1 IgG levels (adjusted r^2^ = 0.073, *p* < 0.001) and anti-IL1α IgG levels (adjusted r^2^ = 0.044, *p* = 0.003). In addition, female patients showed higher anti-IL1β-2 IgG levels than controls (adjusted r^2^ = 0.023, *p* = 0.026). There was no significant change of circulating IgG antibodies to other cytokines. ROC curve analysis showed an AUC of 0.564 for anti-TNFα-1 IgG assay with 22.8% sensitivity against a specificity of 90.0%, and an AUC of 0.539 for anti-IL1α IgG assay with 17.8% sensitivity against a specificity of 90.0%; the anti-IL1β-2 IgG assay had an AUC of 0.580 with 26.3% sensitivity against a specificity of 89.8% in female patients. There was no correlation between plasma IgG levels and carotid intima-media thickness.

**Conclusion:**

Natural antibodies to inflammatory cytokines may be potential biomarkers for atherosclerosis.

## Introduction

Atherosclerosis, the most common cause of cardiovascular diseases, is a chronic and systemic inflammatory disorder mainly affecting the intima of large and medium-sized arteries [1]. Several risk factors are likely to be responsible for the development of atherosclerosis, such as smoking, diabetes mellitus, abdominal obesity, atherogenic dyslipidemia and hypertension [2, 3]. Typically, it has long disease latency and frequently coexists in > 1 vascular bed [4]. This disease can lead to myocardial and cerebral infarctions, stroke as well as coronary heart disease [4, 5]. Nowadays, cardiovascular disease accounts for approximately 30% of all health-related deaths worldwide, making atherosclerotic lesions a common cause of death [6]. Early diagnosis and treatment can slow or halt the worsening of atherosclerosis. It is thus imperative to develop effective methods for the detection of atherosclerosis at an early stage.

Natural autoantibodies in the circulation are emerging as promising diagnostic biomarkers for malignant diseases [7, 8]. Recently, an increase in circulating levels of certain immunoglobulin G (IgG) has been identified in patients with atherosclerosis-related diseases. For instance, Machida et al. reported that circulating levels of auto-antibodies against replication protein A2 (RPA2) were found to be increased in patients with stroke [9]. Elevated levels of antibodies against coatomer protein complex subunit epsilon (COPE) and DAN family BMP antagonist (NBL1) have been suggested to contribute to a high risk of cardiovascular events in patients with obstructive sleep apnea [10, 11]. Therefore, the identification of natural autoantibodies may provide a promising approach for early detection of atherosclerosis-related diseases.

Inflammatory cytokines, including tumor necrosis factor alpha (TNF-α), interleukin (IL) 1α (IL1α), IL1ß, IL-2, IL6 and IL8 are involved in the pathogenesis of atherosclerosis, participating in all stages of this condition [12, 13]. In this study, our main goal was to examine the association between circulating antibody levels against inflammatory cytokines and atherosclerosis, which may identify effective biomarkers for the diagnosis of atherosclerosis.

## Materials and methods

### Subjects

A total of 220 patients diagnosed with atherosclerosis and 200 healthy controls were enrolled from the Second Hospital of Jilin University for this study. Their demographic characteristics are given in Table 1. The inclusion criteria was the presence of abnormal carotid intima-media thickness (CIMT), which is a useful measure for subclinical atherosclerosis and directly associated with an increased risk of both myocardial infarction and ischemic stroke, was measured by vascular ultrasound. Control samples were collected from local communities in the same period as the collection of case samples. Those subjects who had thyroid diseases or other autoimmune diseases such as type-1 diabetes and inflammatory bowel diseases, and those with any type of malignant tumors, were not included for this study. All subjects were of Han Chinese origin and all provided written informed consent to participant in this study. This work was approved by the Ethics Committee of Jilin University Second Hospital and conformed to the Declaration of Helsinki.

**Table 1:** Demographic and clinical characteristics of patients and control subjects

Characteristics	Patients (*n* = 220)	Controls (*n* = 200)	t/χ^2^	*P* value
Gender, n (%)			0.016	0.898
Male	124 (56.6)	112 (56.0)		
Female	96 (43.4)	88 (44.0)		
Age (years)	61.1 ± 11.4	60.8 ± 11.8	0.234	0.815
Site of plaques				
Carotid artery	6 (2.7)	–		
Carotid bifurcation	52 (23.7)	–		
Internal carotid artery	39 (17.8)	–		
Internal carotid bifurcation	2 (0.9)	–		
Common carotid artery	100 (45.7)	–		
Subclavian artery	20 (9.1)	–		

### Antibody testing

Seven linear peptide antigens derived from IL1α, IL1β, IL6, IL8 and TNFα were designed based on computational epitope prediction tools (www.iedb.org) and then synthesized by solid-phase chemical method with a purity of > 95%. The detail of each antigen designed is listed in Table 2. An enzyme-linked immunosorbent assay (ELISA) was developed in-house with these 7 peptide antigens based on previous studies [14]. Briefly, each synthetic peptide antigens was dissolved in 67% acetic acid as a stock solution of 5 mg/ml; a working solution of 20 μg/ml in coating buffer (0.1 m phosphate buffer containing 10 mm EDTA and 0.15 m NaCl, pH 7.2) was used to coat maleimide-activated 96 well microtiter plates (Cat. 15,150, Thermo Scientific, Rockford, IL, USA) based on the manufacturer’s instructions and stored at 4 °C until use. After the antigen-coated microplate was washed twice with 200 μl phosphate-buffered saline (PBS) containing 0.1% Tween-20, 50 μl Assay Buffer (PBS containing 0.5% bovine serum albumin (BSA)) was added to each negative control (NC) well and 50 μl of the diluted plasma and positive control (PC) sample (1100 dilution) in Assay Buffer to the sample wells. After incubation for 1.5 h at room temperature, the plate was washed 3 times with 200 μl Wash Buffer and 50 μl goat anti-human IgG antibody conjugated to peroxidase (ab98567, Abcam, Beijing, China) (1,50,000 dilution in Assay Buffer) was then added. The plate was incubated at room temperature for 1 h, and 50 μl Stabilized Chromogen (SB02, Life Technologies, Guangzhou, China) was used for color development. A microplate reader (BioTek, Winooski, VT, USA) was used to measure the optical density (OD) at 450 nm and a reference wavelength of 620 nm.

**Table 2:** Sequence of peptide antigens used for the in-house ELISA

Antigens	Sequence (N → C)	NCBI accession
IL1α	H-WETHGTKNYFTSVAHPNLFIATKQDYWVC-OH	NP_000566
IL1β-1	H-SLNCTLRDSQQKSLVMSGPYELKALHLQG-OH	NP_000567
IL1β-2	H-KHAYYSGNEDDLFFEADGPKQMKCH-OH	NP_000567
IL6	H-LTKLQAQNQWLQDMTTHLILRSC-OH	NP_000591
IL8	H-DCQCIKTYSKPFHPKFIKELRVIESD-OH	NP_000575
TNFα-1	H-CQLQWLNRRANALLANGVELRDNQLV-OH	NP_000585
TNFα-2	H-KSAIKSPCQRETPEGAEAKPWYEPK-OH	NP_000585

All the samples were assayed in duplicate and relative levels of plasma IgG antibodies were expressed as the specific binding ratio (SBR) that was calculated as follows:

$$ \mathrm{SBR}=\left({\mathrm{OD}}_{\mathrm{Sample}}-{\mathrm{OD}}_{\mathrm{NC}}\right)/\left({\mathrm{OD}}_{\mathrm{PC}}-{\mathrm{OD}}_{\mathrm{NC}}\right). $$

### Data analysis

An inter-assay deviation was estimated by the coefficient of variation (CV) calculated based on the measurement of a pooled plasma sample randomly collected from > 20 healthy subjects, namely quality control (QC) sample that was tested on every plate. Plasma IgG levels were expressed as the mean ± standard deviation (SD) in SBR, and Student’s *t*-test was used to examine the differences between the patient group and the control group. Linear regression analysis was applied to test the effects of disease status on the IgG levels with adjustment for age and gender. Receiver operating characteristic (ROC) curve analysis was performed to assess the value of plasma IgG indicators for the diagnosis of atherosclerosis, with calculation of an area of the ROC curve (AUC) and 95% confidence interval (CI) as well as a sensitivity against the specificity of > 90%. The *p*-value of < 0.05 was considered to be statistically significant. All statistical tests were performed with SPSS 19.0 software (IBM, Armonk, New York).

## Results

All CV calculated with SBR of the QC sample was less than 20% (Table 3), suggesting a good reproducibility for the in-house ELISA. As shown in Table 4, plasma anti-TNFα-1 IgG levels were significantly higher in patients with atherosclerosis patients than control subjects (*t* = 3.588, *p* < 0.001), female patients mainly contributing to increased anti-TNFα-1IgG levels in atherosclerosis (*t* = 3.810, *p* < 0.001). In addition, patients with atherosclerosis had a significantly higher level of circulating IgG against IL1α antigens than control subjects (*t* = 3.084, *p* = 0.002) and female patients mainly contributed to the significant change (*t* = 2.964, *p* = 0.003). While anti-IL1β-2 IgG level was significantly higher than in female patients than female controls (*p* = 0.026), there was no significant difference was found in combined groups.

**Table 3:** Inter-assay deviation between ELISA-testing plates

Factors	Number of plates	Mean ± SD	CV (%)
IL1β-1	20	0.67 ± 0.11	16.94%
IL6	20	1.21 ± 0.15	12.11%
IL8	20	1.28 ± 0.12	9.42%
TNFα-1	20	1.24 ± 0.07	5.87%
IL1α	20	0.93 ± 0.07	7.45%
IL1β-2	20	1.14 ± 0.10	9.12%
TNFα-2	20	1.79 ± 0.29	16.10%

*SD* standard deviation, *CV* coefficient of variation

**Table 4:** Differences of circulating IgG between the two groups

Factors	Atherosclerosis (Mean ± SD)	Control (Mean ± SD)	*t*	*P* value
IL1β-1				
Male	0.651 ± 0.310 (124)	0.657 ± 0.358 (112)	−0.138	0.890
Female	0.674 ± 0.331 (96)	0.634 ± 0.419 (88)	0.709	0.480
Total	0.661 ± 0.319 (220)	0.647 ± 0.385 (200)	0.399	0.690
IL6				
Male	1.204 ± 0.357 (124)	1.188 ± 0.350 (112)	0.353	0.725
Female	1.297 ± 0.437 (96)	1.199 ± 0.545 (88)	1.349	0.179
Total	1.244 ± 0.396 (220)	1.193 ± 0.445 (200)	1.259	0.209
IL8				
Male	1.087 ± 0.300 (124)	1.092 ± 0.295 (112)	−0.105	0.916
Female	1.145 ± 0.292 (96)	1.101 ± 0.307 (88)	1.007	0.315
Total	1.113 ± 0.298 (220)	1.096 ± 0.300 (200)	0.581	0.561
TNFα-1				
Male	1.198 ± 0.296 (124)	1.145 ± 0.265 (112)	1.438	0.152
Female	1.291 ± 0.302 (96)	1.133 ± 0.253 (88)	3.810	**<0.001**
Total	1.238 ± 0.301 (220)	1.140 ± 0.259 (200)	3.588	**<0.001**
IL1α				
Male	0.883 ± 0.162 (124)	0.852 ± 0.159 (112)	1.490	0.138
Female	0.906 ± 0.148 (96)	0.835 ± 0.176 (88)	2.964	**0.003**
Total	0.893 ± 0.156 (220)	0.845 ± 0.166 (200)	3.084	**0.002**
IL1β-2				
Male	1.008 ± 0.201 (124)	1.008 ± 0.233 (112)	0.031	0.975
Female	1.085 ± 0.270 (96)	1.002 ± 0.224 (88)	2.248	**0.026**
Total	1.042 ± 0.236 (220)	1.005 ± 0.229 (200)	1.603	0.110
TNFα-2				
Male	1.385 ± 0.427 (124)	1.457 ± 0.497 (112)	−1.195	0.233
Female	1.486 ± 0.426 (96)	1.492 ± 0.431 (88)	−0.090	0.928
Total	1.429 ± 0.428 (220)	1.472 ± 0.468 (200)	−0.991	0.322

*P* < 0.05

*P* value in bold was statistically significant

As shown in Table 5, linear regression analysis showed that atherosclerotic patients had an increase in circulating anti-TNFα-1 IgG levels (adjusted *r*^2^ = 0.038, *p* < 0.001) and anti-IL1α IgG level (adjusted *r*^2^ = 0.025, *p* = 0.002) compared with control subjects, female patients mainly contributing to increased anti-TNFα-1 IgG levels (adjusted r^2^ = 0.073, *p* < 0.001) and increased anti-IL1α IgG levels (adjusted *r*^2^ = 0.044, *p* = 0.003). Additionally, female patients had an increase in anti-IL1β-2 IgG levels compared to female controls (adjusted *r*^2^ = 0.023, *p* = 0.026).

**Table 5:** Multivariate linear regression analysis of circulating IgG against cytokines in atherosclerosis

Factors	Adjusted R^2^	*P* value*	β & 95% CI
IL1β-1			
Male	−0.001	0.866	−0.007 (− 0.093, 0.078)
Female	−0.008	0.481	0.039 (−0.071, 0.150)
Total	−0.004	0.698	0.013 (−0.054, 0.081)
IL6			
Male	−0.006	0.736	0.016 (−0.075, 0.107)
Female	0.003	0.179	0.098 (−0.045, 0.242)
Total	0.001	0.204	0.052 (−0.029, 0.133)
IL8			
Male	−0.009	0.915	−0.004 (− 0.081, 0.073)
Female	0.003	0.316	0.045 (− 0.043, 0.132)
Total	−0.001	0.564	0.017 (−0.041, 0.074)
TNFα-1			
Male	0.012	0.161	0.051 (−0.021, 0.124)
Female	0.073	**< 0.001**	0.157 (0.076, 0.239)
Total	0.038	**< 0.001**	0.098 (0.044, 0.152)
IL1α			
Male	0.012	0.146	0.030 (−0.011, 0.071)
Female	0.044	**0.003**	0.071 (0.024, 0.118)
Total	0.025	**0.002**	0.048 (0.017, 0.079)
IL1β-2			
Male	0.009	0.988	−0.0004 (−0.056, 0.055)
Female	0.023	**0.026**	0.083 (0.010, 0.155)
Total	0.017	0.111	0.036 (−0.008, 0.080)
TNFα-2			
Male	0.012	0.215	−0.074 (− 0.192, 0.044)
Female	−0.011	0.928	−0.006 (− 0.131, 0.120)
Total	0.006	0.316	−0.044 (− 0.130, 0.042)

*Adjusted *P*-value for age in male and female samples, and for gender and age in combined samples; *CI* confidence interval

**Fig. 1:**
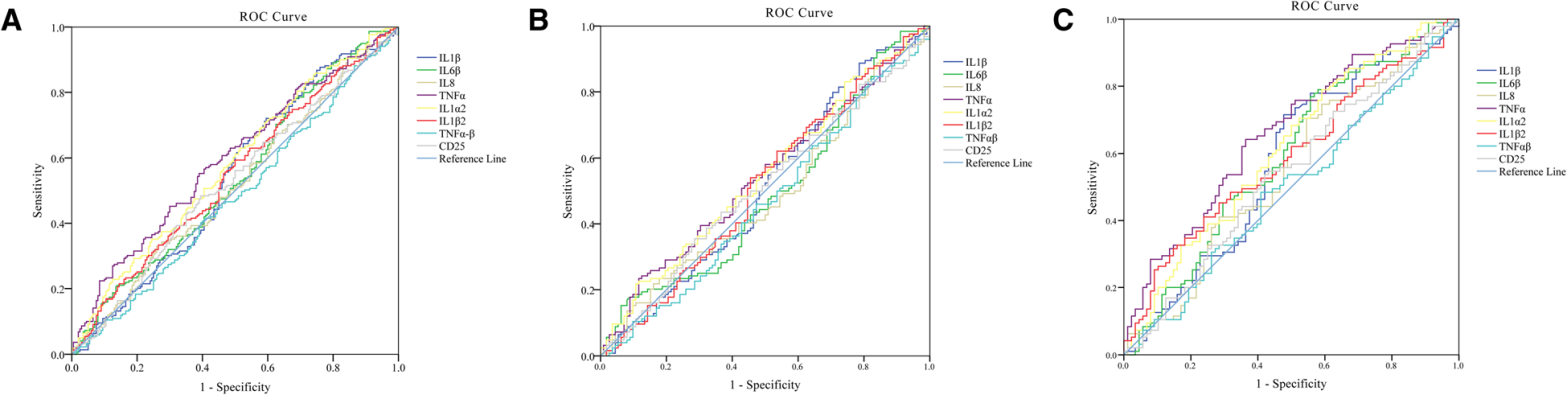
ROC curve analysis of circulating IgG in atherosclerosis. **a** combined subjects; (**b**) male subjects; (**c**) female subjects

ROC curve analysis revealed an AUC of 0.564 (95% CI0.509–0.619) for anti-TNFα-1 IgG assay with 22.8% sensitivity against the specificity of 90.0%, and an AUC of 0.539 (95% CI 0.484–0.594) for anti-IL1α IgG assay with17.8% sensitivity against the specificity of 90.0%. Moreover, ROC curve analysis performed only in females showed that anti-TNFα-1 IgG assay had an AUC of 0.591 (95% CI 0.509–0.673) with 28.4% sensitivity against a specificity of 89.8%, and anti-IL1α IgG assay had an AUC of 0.549 (95% CI 0.466–0.632) with 20.0% sensitivity against a specificity of 89.8%, and anti-IL1β-2 IgG assay had an AUC of 0.580 (95% CI 0.498–0.663) with 26.3% sensitivity against a specificity of 89.8% (Fig. 1).

As shown in Table 6, there was no correlation between plasma IgG levels and CIMT.

**Table 6:** The correlations between plasma IgG levels and CIMT

Factors	df	Coefficients of correlation	*P* value
IL1β-1	218	0.053	0.432
IL6	218	0.007	0.920
IL8	218	0.060	0.379
TNFα-1	218	0.003	0.968
IL1α	218	0.058	0.397
IL1β-2	218	−0.081	0.234
TNFα-2	218	−0.064	0.347

## Discussion

Recent studies have demonstrated the presence of natural autoantibodies in blood of patients with atherosclerosis, such as anti-apolipoprotein A-1 antibodies and anti-lipoprotein lipase antibodies [15, 16]. TNFα induces a pro-inflammatory process in endothelial cells, altering function of endothelial and vascular smooth muscle cells, which is crucially involved in the progression of atherosclerosis and heart failure [17, 18]. A study suggests that the role of IL-1α in atherogenesis should be targeted in patients with cardiovascular disease [19]. Decreased IL1β level was found to be related to the inhibition of platelet aggregation and thromboembolic-related disorders [20]. In this study, we found that plasma anti-TNFα and anti-IL1α IgG levels were significantly increased in patients with atherosclerosis compared with control subjects, and an increase in anti-IL1β IgG level was found in female patients (Table 4). Although circulating levels of anti-TNFα and anti-IL1α IgG antibodies were significantly increased in atherosclerosis, ROC curve analysis revealed relatively low sensitivity (Fig. 1). Possibly, such an antibody test cannot serve as highly effective biomarkers for diagnosis of the disease but represent a subgroup of atherosclerotic patients who may have developed chronic inflammation in their body. Nevertheless, the findings suggest that natural antibodies against inflammatory cytokines such as TNFα, IL1α, and IL1β may serve as useful biomarkers for the identification of atherosclerotic subgroup that may need immunological treatment although whether the levels of these inflammatory cytokines are correlated with their antibody levels in the circulation need further investigation.

Several studies have indicated gender differences in the development of atherosclerosis [21, 22]. Androgens could up-regulate the expression of atherosclerosis-related genes in macrophages from males but not females, suggesting genetic predisposition to atherosclerosis only in male subjects [23]. Knowledge into biological differences in atherosclerosis between men and women remains incomplete. In this study, the gender differences in circulating IgG antibodies to inflammatory cytokines were observed, so that up-regulation of anti-TNFα, anti-IL1α, and anti-IL1β IgG levels were more likely to occur in female than male patients with atherosclerosis. Collectively, the gender differences in circulating IgG antibodies to inflammatory cytokines may provide a clue to insights into the pathophysiology of atherosclerosis.

Technically, ELISA antibody tests with individual antigens may have a relatively low sensitivity as shown in this study. Possibly, such an antibody test alone is unlikely to screen people with atherosclerosis in clinical practice. Several studies have demonstrated that a panel of cancer-associated antigens had a high sensitivity for early detection of cancer [24, 25]. Future work on identification of a panel of such linear peptide antigens may provide an aid to early screening of atherosclerosis.

## Conclusions

Natural antibodies to inflammatory cytokines may be potential biomarkers for atherosclerosis although further replication with a large sample size is needed to confirm this initial finding.

## Abbreviations

AUC: Areas under the ROC curve
BSA: Bovine serum albumin
CI: Confident interval
CIMT: Carotid intima-media thickness
CV: Coefficients of Variation
ELISA: Enzyme-linked immunosorbent assay
IgG: Immunoglobulin G
IL1α: Interleukin 1α
IL1β-1: Interleukin 1β derived antigen 1
IL1β-2: Interleukin 1β derived antigen 2
IL6: Interleukin 16
IL8: Interleukin 8
NC: Negative control
OD: Optical density
PBS: Phosphate -buffered saline
PC: Positive control
QC: Quality control
ROC: Receiver operating characteristic curve
RPA2: Replication protein A2
SBR: Specific binding ratio
TNFα-1: Tumor Necrosis Factor α derived antigen 1
TNFα-2: Tumor Necrosis Factor α derived antigen 2
WHO: World Health Organization
